# Supplementary material for: From sequence to enzyme mechanism using multi-label machine learning
Source: BMC Bioinformatics. 2014 May 19;15:150. doi: 10.1186/1471-2105-15-150 (PMC4229970; doi:10.1186/1471-2105-15-150)
Supplement: Additional file 2 — Java code of ml2db. Additional file ml2db_code.tar.gz contains the Java source code to run the multi-label machine learning experiments and save the results to database. The code’s Javadoc is included. [file 1471-2105-15-150-S2.zip › additional file 2/ml2db/ml2db/doc/index-files/index-10.html]

J-Index


JavaScript is disabled on your browser.


- Overview
- Package
- Class
- Use
- Tree
- Deprecated
- Index
- Help

- Prev Letter
- Next Letter

- Frames
- No Frames

- All Classes

A B C D E F G H I J K L M N O P Q R S T U V W X 


## J

JaccardDistance - Class in main.ml2db.weka
:   This is an implementation of the Jaccard distance which calculates it on the
    fly.

JaccardDistance() - Constructor for class main.ml2db.weka.JaccardDistance


JaccardDistance(Instances) - Constructor for class main.ml2db.weka.JaccardDistance


JaccardDistanceStored - Class in main.ml2db.weka
:   This is an implementation of the Jaccard distance which pre-calculates and
    stores the numbers needed.

JaccardDistanceStored(IdMultiLabelInstances) - Constructor for class main.ml2db.weka.JaccardDistanceStored


JaccardDistanceTest - Class in main.ml2db.weka


JaccardDistanceTest() - Constructor for class main.ml2db.weka.JaccardDistanceTest


JaccardEuclideanDistance - Class in main.ml2db.weka
:   A Jaccard distance.

JaccardEuclideanDistance() - Constructor for class main.ml2db.weka.JaccardEuclideanDistance


joinInstances(String, String) - Method in class main.ml2db.al.utility.globaldistance.DistancesStorage
:   Beware, it strips first and last character from the instance before
    joining! (if divider is pipe) From {0 1,1 1,2 1}, {3 1,4 1} to 0 1,1 1,2
    1|3 1,4 1

JPG - Static variable in class main.ml2db.graphviz.GraphViz

A B C D E F G H I J K L M N O P Q R S T U V W X

- Overview
- Package
- Class
- Use
- Tree
- Deprecated
- Index
- Help

- Prev Letter
- Next Letter

- Frames
- No Frames

- All Classes
